# Endometrial Dysbiosis Is Related to Inflammatory Factors in Women with Repeated Implantation Failure: A Pilot Study

**DOI:** 10.3390/jcm11092481

**Published:** 2022-04-28

**Authors:** Vito Cela, Simona Daniele, Maria Elena Rosa Obino, Maria Ruggiero, Elisa Zappelli, Lorenzo Ceccarelli, Francesca Papini, Ilaria Marzi, Giorgia Scarfò, Fulvia Tosi, Ferdinando Franzoni, Claudia Martini, Paolo Giovanni Artini

**Affiliations:** 1 Division of Gynecology and Obstetrics, Department of Clinical and Experimental Medicine, University of Pisa, 56100 Pisa, Italy; celav2001@gmail.com (V.C.); mariaelena.obino@gmail.com (M.E.R.O.); papinifra@libero.it (F.P.); ilaria.marzi@ao-pisa.toscana.it (I.M.); 2 San Rossore Clinic Care, 56100 Pisa, Italy; fertility@sanrossorecura.it (M.R.); f.tosi@sanrossorecura.it (F.T.); 3 Department of Pharmacy, University of Pisa, 56100 Pisa, Italy; e.zappelli@studenti.unipi.it (E.Z.); lorenzo.ceccarelli@phd.unipi.it (L.C.); claudia.martini@unipi.it (C.M.); 4 Department of Biotechnology, Chemistry, and Pharmacy, University of Siena, 53100 Siena, Italy; 5 Division of General Medicine, Department of Clinical and Experimental Medicine, University of Pisa, 56100 Pisa, Italy; g.scarfo1@studenti.unipi.it (G.S.); ferdinando.franzoni@unipi.it (F.F.)

**Keywords:** endometrial microbiota, dysbiosis, inflammation, in vitro fertilization, repeated implantation failure

## Abstract

An abnormal endometrial microbiota has been suggested to impair the process of embryo implantation, thus leading to repeated implantation failure (RIF) in women undergoing in vitro fertilization (IVF). However, the molecular mechanisms linking uterine microbiota and IVF out-comes are still an open question. The aim of this cohort study was to outline the relationship between endometrial microbiota, inflammation and IVF outcomes. To this purpose, endometrial microbiota and selected components of the “cytokine network” were analyzed in women presenting RIF and divided between eubiosis and dysbiosis groups, according to the percentage of endometrial lactobacilli (≥90% or <90%, respectively). The Dysbiosis group presented significantly higher tissue concentrations of the inflammatory markers (IL-6, IL-1β, HIF-1α and COX-2) and significantly lower levels of the anti-inflammatory/well-being factors, IL-10 and IGF-1, with respect to women with eubiosis. Moreover, the Lactobacillus percentage was negatively related to the concentrations of the inflammatory molecules and positively related to IL-10/IGF-1. Interestingly, the number of IVF attempts was directly related to the levels of the inflammatory factors COX-2, IL-1β and HIF-1α in the eubiosis group. Overall, endometrial dysbiosis was demonstrated to be associated with inflammation-related endometrial changes affecting the process of embryo implantation, underlining the importance of assessing uterine microbiota in patients undergoing IVF.

## 1. Introduction

The uterine cavity had been considered to be sterile until recent studies revealed the existence of a specific endometrial microbiota dominated by *Lactobacilli* [1,2] and to a lesser extent by *Actinomyces*, *Bifidobacterium*, *Propionibacterium*, *Staphylococcus*, and *Streptococcus* genera [1]. Further studies have identified a different microbial composition in the cervical canal, uterus, fallopian tubes, peritoneal fluid and the vagina [3], pointing out that the maintenance of this exact bacteria distribution is necessary in order to prevent pathologies of the reproductive system, including uterine inflammation [4], endometriosis [5] or endometrial cancer [6].

In this sense, the microbial composition of the female reproductive tract has been associated with pathological shifts affecting the reproductive process, including fertilization, implantation, maintenance of pregnancy and microbial colonization of the newborn [7,8,9]. Uterine microbiota constitutes an essential aspect of the reproductive tract and is characterized by Firmicutes, Bacteroidetes, Proteobacteria and Actinobacteria, whose equilibrium is pivotal for maintaining a microenvironment modulating host immunity and uterine health [7]. Thus, a fertile endometrium benefits from the microbial contribution to physiological processes, including endometrial receptivity, highlighting the association between the stability, composition of the uterine microbiota and the success of the reproductive process [2,7]. The current literature underlies a strict association between uterine microbial composition and the success of the reproductive process [2,7]. In particular, several reports have suggested that human uterine microbiota is involved in the process of embryo implantation. Specifically, previous studies by Moreno and collaborators have demonstrated that the presence of at least 90% of *Lactobacillus* in the endometrial fluid is beneficial for embryo implantation in IVF-embryo transfer (ET) in the infertile women. Conversely, the presence of non-Lactobacillus-dominated microbiota is related with significant decrease in implantation, pregnancy, ongoing pregnancy, and live birth rates [1,2]. In this sense, the presence of bacterial pathogens such as *Mycoplasma*, *Ureaplasma* and *Gardnerella* [10], rather than *Lactobacillus* species, has been associated to chronic endometritis (CE), a pathological condition characterized by a persistent inflammation of the endometrial mucosa, which is often asymptomatic and ignored in the clinic [11,12,13]. In addition, up to 40% of patients with repeated implantation failure (RIF) presented CE, often with a delayed diagnosis, thus highlighting an association between CE, chronic inflammation and pregnancy failure. The dysbiotic state of CE can impair entailed inflammation and immune activation in the endometrium, thus impairing endometrial receptivity. In particular, immune cells have been speculated to alter decidualization and trigger cellular mechanisms, including endometrial proliferation, autophagy and uterine contraction, which in turn impair endometrial receptivity [7,14]. Furthermore, a non-*Lactobacillus*-dominated microbiota and the presence of pathogens have been linked to an alteration of the “cytokine network” [15]. In this sense, tumor factor necrosis alpha (TNF-α), interferon gamma (IFN-γ) and cytokine IL-2 have been suggested to inhibit implantation in mouse models [16], whereas interleukins IL-10 and IL-4 are essential for implantation and trophoblast development [17].

Nevertheless, the association between inflammation-related dysbiosis and RIF and the molecular mechanisms linking endometrial eubiosis to embryo implantation in IVF procedures remain to be consolidated in human subjects. The investigations on the uterine microbiota have been limited by vaginal contamination and low biomass [7], and just a few studied have investigated the association between endometrial microbiota composition/alteration and the IVF outcomes in depth. Herein, endometrial microbiota was examined in infertile women presenting RIF: selected components of the cytokine network were examined in the same endometrial biopsies to outline the relationship between uterine microbiota, inflammation and IVF outcomes. Considering the high incidence of infertility and the existence of RIF in IVF treatments, determining the link between endometrial dysbiosis and infertility related to endometrial receptivity is of pivotal importance to provide insights on modulating uterine receptivity, thus managing protocols during IVF cycles. Indeed, while technological advances in IVF techniques have led to increase fertilization rates, pregnancy rates have remained fairly unchanged, suggesting that further understanding of processes related to endometrial receptivity is of absolute need [18].

## 2. Materials and Methods

### 2.1. Patients

In this cohort study, patients (N = 26) aged between 33 and 42 years, undergoing IVF treatment at the Centre for Infertility and Assisted Reproduction of the Department of Clinical and Experimental Medicine of Pisa, were recruited. All patients underwent a complete clinical history and physical examination, biochemical analyses and transvaginal ultrasonography. The study was conducted according to the guidelines of the Declaration of Helsinki and approved by the Ethics Committee (CTO, Clinical Trial Office) of Azienda Ospedaliero Universitaria Pisana (AOUP) (protocol code 35105, approved on 13 June 2019).

The patients underwent IVF treatment and biopsies sampling at the Centre of Infertility and Assisted Reproduction of the Department of Clinical and Experimental Medicine of Pisa, between May 2019 and April 2020. All women had been proven to have primary infertility for at least 3 years. The following inclusion criteria were applied: (1) three or more failed embryo transfer with good-quality embryos; (2) had not used antibiotics or vaginal medications and probiotics for more than 1 month; (3) had not had a previous diagnosis of endometritis. Women with unexplained primary infertility (N = 16) or tubal factor (N = 10) were included in the study. The exclusion criteria included the following: (1) uterine cavity diseases, including polyps, endometriosis, fibroids, adenomyosis and cancer’ (2) ovarian tumor, fallopian tube effusion, abnormal female hormones or unexplained bleeding; (3) abnormal vaginal discharge examination (4) suspected pelvic inflammatory disease; (5) endocrinological disorder, including thyroiditis or polycystic ovarian syndrome or immunological disorders; (6) secondary infertility; (7) varicocele or azoospermia of the male partner: couples undergoing ICSI were excluded from the study; (8) smoking; (9) women with obesity.

**Figure 1:**
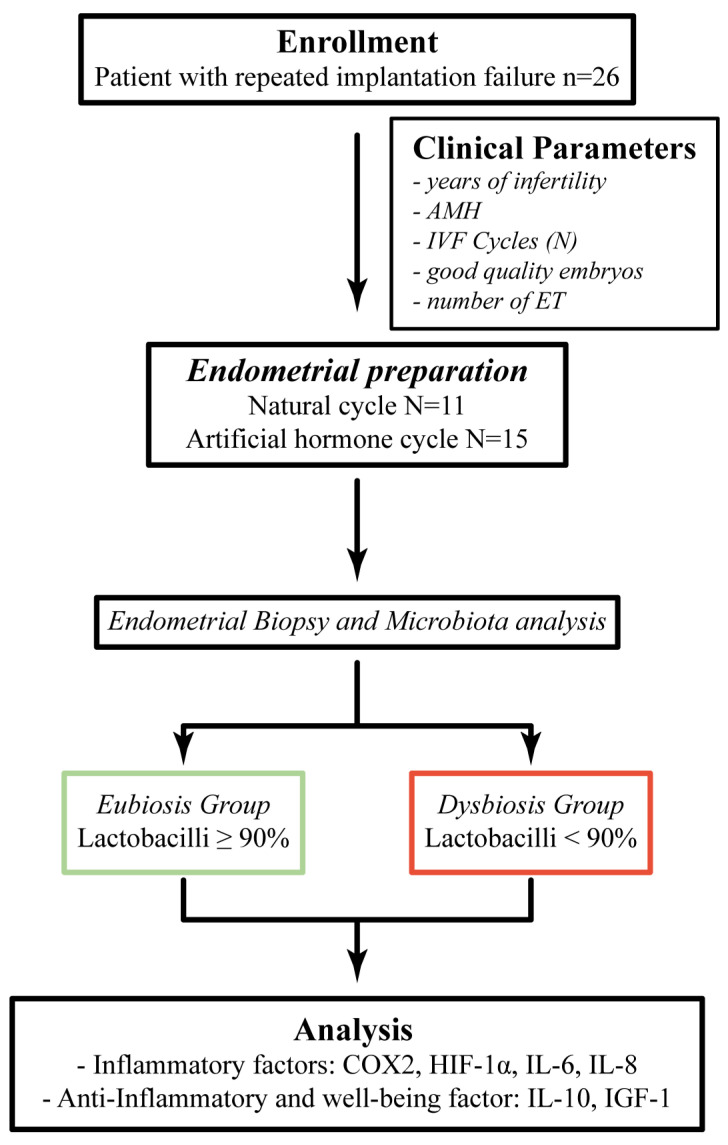
Schematic overview of the clinical workflow. Good quality embryos, number of IVF cycles, number of embryo-transfer (ET) and good quality embryos/number of trials are expressed as median values. AMH: anti-Mullerian hormone.

All patients underwent endometrial preparation to reach a sufficient endometrium thickness, and then underwent endometrial biopsy to quantify the amount of lactobacilli present. Based on the percentage of lactobacilli, the patients were divided into two groups: the group defined as *Eubiosis*, including patients who had a percentage of lactobacilli ≥90%; values <90% were considered as conditions of endometrial *Dysbiosis* [2]. Based on this subdivision, inflammatory and well-being mediators were quantified by specific commercial immunoenzymatic assays. The clinical workflow related to the present study is represented in Figure 1.

### 2.2. Sample Collection

Endometrial biopsy samples were obtained on 18–22 days (natural cycles, N = 11) or on progesterone + 5 days (artificial hormone cycle, N = 15). These two protocols (i.e., natural and artificial hormone cycles) were comparable in reaching a sufficient endometrial thickness for the biopsy collection.

The tissue collection was performed according to the instructions for endometrial microbiome genomic analysis in order to avoid possible contamination and degradation of the genetic material. In particular, after removing the mucous membranes, the vaginal cavity and cervix were cleaned twice using sterilized cotton balls soaked in benzalkonium chloride solution. An endosuction was carefully inserted into the uterus to avoid contact with the vulva and vaginal walls, and endometrial tissue was collected by a MedGyn IV pipette (MedGyn, Addison, IL, USA). The collected biopsies were stocked at −80 °C until processing for DNA extraction and protein analyses.

### 2.3. Bacterial DNA Extraction and RT-PCR

Endometrial biopsies were processed to extract total DNA and then to determine the microbiota composition, in terms of percentage of lactobacilli and of the microbial species reported in Table 1. In detail, the total bacterial DNA was extracted from endometrial tissue samples using the QIAamp^®^ DNA Microbiome Kit (QIAGEN, Hilden, Germany) according to the manufacturer’s protocol [19]. The extracted DNA was quantified using NanoDrop (Thermofisher, Waltham, MA, USA) and amplified with MiniOpticon (BIORAD, Milano, Italy). RT-PCR reactions consisted of 10 μL of Fluocycle^®^ II SYBR^®^ (Euroclone, Milan, Italy), 0.6 μL of 10 μM forward and reverse primers, 5 μL of cDNA and 3.8 μL of H_2_O. All reactions were performed for 40 cycles using the following temperature profiles: 98 °C for 30 s (initial denaturation); T °C (see Table 1) for 30 s (annealing); and 72 °C for 3 s (extension). The specificity of PCR was also determined by gel electrophoresis. In order to identify the correct primers for each bacterial species, a bibliographic search was performed (Table 1). The chosen primers were re-evaluated and subjected to the Basic Local Alignment Search Tool (BLAST) which, using the Genomic database of the National Centre for Biotechnology Information, searches for local sequence homologies and returns a series of sequences accompanied by two columns of values certifying the goodness of alignments. All primers were purchased from Sigma Aldrich (Milan, Italy).

### 2.4. Quantification of Absolute and Relative Bacterial DNA

The bacterial DNA content derived from the Lactobacillus (Microtec Cagliari) genus was analyzed. The primers used are summarized in Table 1. A panel of DNA models of bacteria responsible for chronic endometritis was selected to evaluate the specificity of the RT-PCR tests [19]. A total of 14 DNA templates were obtained from Deutsche Sammlung von Mikroorganismen und Zellkulturen (DSMZ, Braunschweig, Germany) or from Belgian Coordinated Collections of Micro-organisms (BCCM/LMG, Gent, Belgio), including templates of common microorganisms of the disease, Enterobacteriaceae species (LMG 2783), Enterococcus species (LMG 17117), Escherichia coli (LMG 2092), Gardnerella vaginalis (LMG 7832), Klebsiella pneumoniae (LMG 2095), Mycoplasma hominis (DSMZ 106704), Staphylococcus species (DSMZ 1798), Streptococcus species (LMG 8518), Pseudopropionibacterium rubrum (DSMZ 100122), Neisseria Subflava (LMG 5313), and Prevotella (DSMZ 15606), as well as templates of sexually transmitted disease pathogens, Chlamydia trachomatis (DSMZ 19440) and Neisseria gonorrhoeae (DSMZ 9188). The absolute amount of total bacterial DNA was determined by a real time-PCR (RT-PCR) using a calibration curve, *CT* = f (log10 [pgDNA]), generated with known concentration of a standard bacterial DNA and primer for the conserved region of the bacterial 16S rRNA gene (Table 1). The calculated amount of bacterial DNA was used to determine a “correction factor (CF)” for each saliva sample. This CF made it possible to calculate the exact amount of bacterial DNA with respect to the amount of DNA measured with the NanoDrop, which was found to contain a non-negligible amount of human host DNA. The CF indicates the bacterial DNA fraction relative to the total DNA extracted with the kit.

The absolute amount of bacterial DNA derived from each microorganism was also determined by RT-PCR using a calibration curve generated for each specific primer pair. In these cases, the real amount of bacterial DNA loaded in the RT-PCR mix was calculated by multiplying the total DNA loaded by the CF and the result was used to calculate the relative amount of DNA derived from each microorganism to the total bacterial DNA.

### 2.5. Interleukins (ILs) in Endometrial Biopsy

The same endometrial biopsies collected for microbiota determination were employed for the quantification of inflammatory and anti-inflammatory molecules. All the interleukins were measured using enzyme-linked immunosorbent assay (ELISA) kits (Cloud-Clone Corp., Katy, TX, USA: SEA563Hu IL-1β, SEA079Hu for IL-6, SEA080Hu for IL-8 and SEA056Hu for IL-10) following the manufacturers’ instructions. Briefly, 100 µL of endometrial biopsy supernatant and of each dilution of standard was added into the appropriate wells and incubated for 1 h at 37 °C. After incubation time, 100 µL of primary antibody were added for 1 h at 37 °C. After three washes, 100 µL of secondary antibody were incubated for 30 min at 37 °C and then, the substrate solution was added to each well, leaving the color to develop for 10–20 min at 37 °C. Absorbance was measured at 450 nm. 

### 2.6. COX2, HIF-1α and IGF-I Quantification in Endometrial Biopsy

COX2, HIF-1α and IGF-I levels were quantified using an enzyme-linked immunosorbent assay (ELISA) kit (Sigma-Aldrich, Milano, Italy: RAB1034, RAB1057-1and RAB0228, respectively) following the manufacturers’ instructions. Briefly, 100 µL of each standard and sample were incubated for 2.5 h at room temperature with gentle shaking. After four washes with 200 µL of wash buffer 1×, 100 µL of 1× detection antibody were incubated for 1 h at room temperature with gentle shaking. After the completed incubation time, 100 µL of prepared Streptavidin solution was added to each well and incubated for 45 min at room temperature. At the end, TMB One-Step Substrate Reagent was added to each well, leaving the color to develop for 10–20 min at 37 °C. Absorbance was measured at 450 nm. 

### 2.7. Statistical Analysis

The GraphPad Prism (GraphPad Software Inc., San Diego, CA, USA) was used for data analysis and graphical presentations. The data are presented as the mean ± SD or median, as indicated. Statistical analyses were performed by the unpaired *t*-test or Mann–Whitney for normal and non-normal distributed data, respectively.

Correlation between parameters (i.e., correlations between microbiota composition, biochemical markers and clinical parameters related to the Total population and the eubiosis and dysbiosis subjects) was determined by simple linear regression analysis, using the StatView program (Abacus Concepts, Inc., SAS Institute, Cary, NC, USA). The analyses will be considered significant with a 95% confidence interval (CI) from the mean and a p level of 0.05. A sample size calculator (G* power analysis) on the primary outcome (inflammation in endometrial dysbiosis and RIF) was conducted to evaluate the accuracy of the results: accordingly, the study group required 24 patients to obtain the same difference with α = 0.05 and statistical power of 85%.

## 3. Results

### 3.1. Descriptive Statistics

Patients undergoing IVF treatments and presenting RIF were enrolled in the present study (N = 26). The main clinical characteristics and IVF outcome parameters of patients are depicted in Table 2. Women were divided in the eubiosis (N = 13) or dysbiosis (13) group according to the percentage of lactobacilli present in the endometrial biopsy, as reported below. There were no cigarette smokers and obese women (body mass index > 30) in both groups. Mean age, the percentage of occupation, BMI, years and type of infertility did not differ among the two groups. Moreover, the number of good-quality embryos and the numbers of embryo-transfer were found to be comparable between the enrolled groups (Table 2).

### 3.2. Determination of Microbiota Composition in Patient’s Biopsies

**Figure 2:**
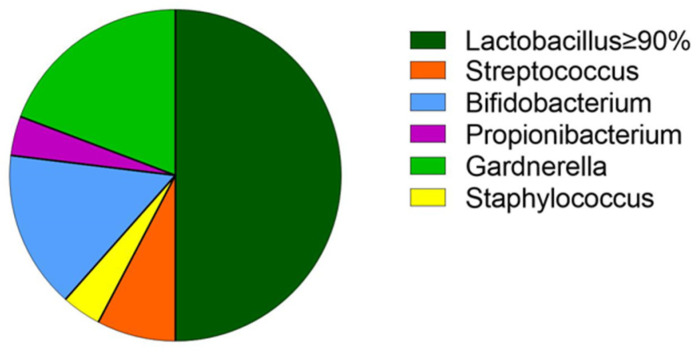
Subdivision of the bacterial population in the two groups. The green slice shows the percentage of the population in eubiotic conditions, while the remainder represents the population of bacteria in patients with dysbiotic conditions.

A real time-PCR analysis was performed to determine the relative abundance of selected microbial species in the collected biopsies (Figure 2; Table 3). In particular, frozen endometrial specimens were assessed for the presence of lactobacilli and chronic endometritis pathogens by RT-PCR using specific primers for the most common bacteria responsible for causing chronic endometritis (*C. trachomatis*, *Enterococcus species*, *E. coli*, *G. vaginalis*, *Klebsiella pneumoniae*, *M. hominis*, *N. gonorrhoeae*, *Staphylococcus species*, and *Streptococcus species*) [13].

As stated above, the enrolled population was divided into eubiosis (N = 13) or dysbiosis (N = 13) groups according to the percentage of lactobacilli present in the endometrial biopsy (eubiosis: lactobacilli ≥= 90%, dysbiosis: lactobacilli <90%) (Figure 2). Of note, lactobacilli species were totally absent in three women. Among dysbiotic patients, the most abundant pathogens were ascribed to Streptococcus (N = 6) or Gardnerella (N = 5) genera (Table 3 and Figure 2).

By covariate analysis, our data evidenced an inverse correlation between Lactobacillus and Streptococcus abundances (*p* = 0.0225), even with a low number of patients presenting this pathogen (N = 6). As expected, the percentage of lactobacilli was not significantly related to Bifidobacterium (*p* = 0.0903).

### 3.3. Inflammatory and Anti-Inflammatory Molecules in Endometrial Biopsies

**Figure 3:**
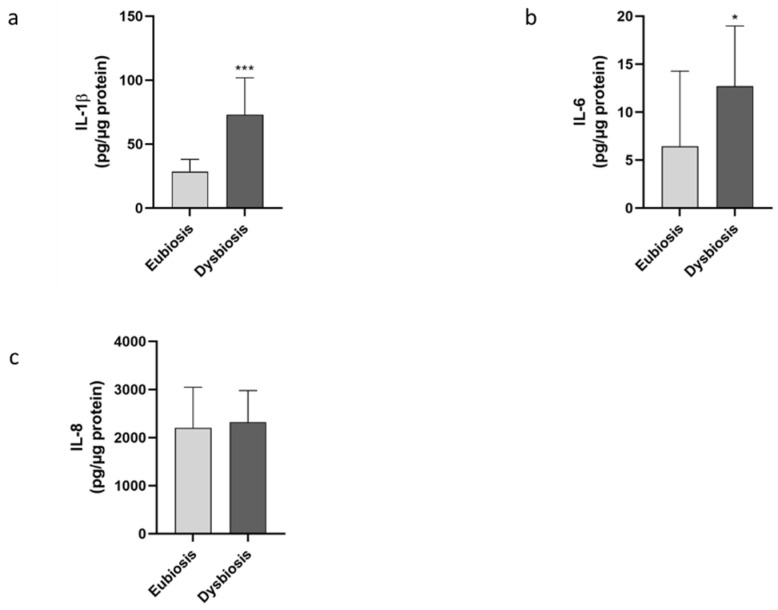
Evaluation of inflammatory molecules. The inflammatory markers were quantified in the two different groups by specific immunoenzymatic assay. In particular, the levels of IL-6 (**a**), IL-1β (**b**) and IL-8 (**c**) were analyzed. The data are the mean ± SEM of three different experiments, each performed in duplicate. Statistical analysis was performed by unpaired *t*-test: * *p* < 0.05, *** *p* < 0.001 vs. eubiosis group.

In order to explore the interplay between dysbiosis and the presence of an altered endometrial microenvironment, a panel of inflammatory markers were quantified in the same endometrial biopsies. Specifically, pro-inflammatory interleukins (IL-1β, IL-6 and IL-8) and factors (i.e., hypoxia-inducible factor 1, HIF-1α and COX-2) were quantified in biopsies of patients. Moreover, the levels of the anti-inflammatory factor IL-10 and IGF-1 were analyzed as putative indicators of endometrial wellness. The mean values of the measured parameters in the two groups are reported in Table 4. The pro-inflammatory ILs, IL-6 (*p* = 0.0238) and IL-1β (*p* < 0.0001), exhibited significantly higher levels in the dysbiosis group than in the eubiosis one (Figure 3a,b). In contrast, no significant variation was noticed for IL-8 concentration in the two groups (*p* = 0.9093, Figure 3c).

**Figure 4:**
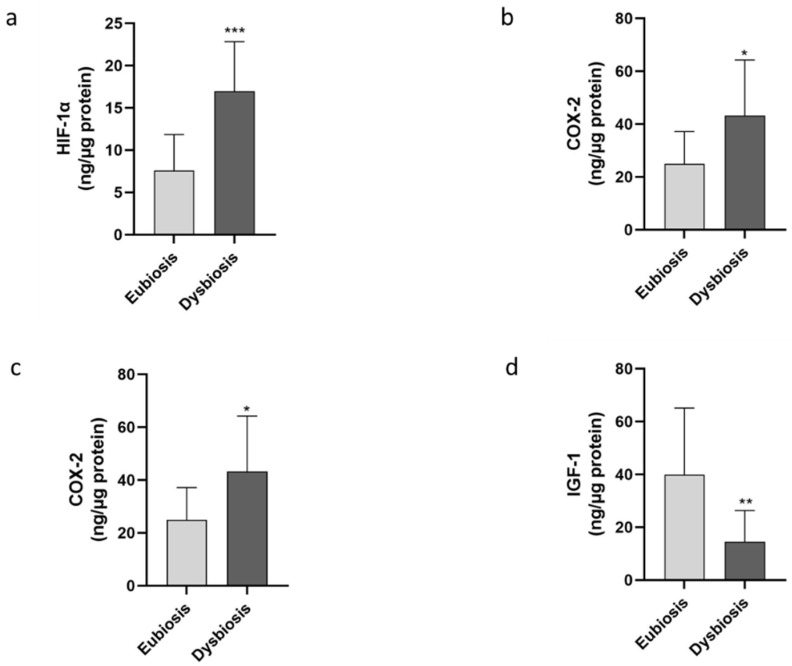
Evaluation of inflammatory and anti-inflammatory molecules. The inflammatory markers were quantified in the two different groups by specific immunoenzymatic assay. In particular, the levels of HIF-1α (**a**), COX-2 (**b**), IL-10 (**c**) and IGF-1 (**d**) were analyzed. The data are the mean ± SEM of three different experiments, each performed in duplicate. Statistical analysis was performed by unpaired *t*-test: * *p* < 0.05, ** *p* < 0.01, *** *p* < 0.001 vs. eubiosis group.

A harmful microbiota has been related to the production of toxins which induce an increase in the hypoxia-inducible factor 1 (HIF-1α) [20]. Consistent with these data, our results demonstrated a significant increase in HIF-1α levels in the group of patients with endometrial dysbiosis with respect to eubiotic patients (*p* = 0.0002, Figure 4a). Furthermore, the expression of Cyclooxygenase-2 (COX-2), an enzyme associated with inflammation, was significantly elevated in patients with dysbiosis than in the eubiosis group (*p* = 0.0060, Figure 4b). Consistent with a general enhancement in pro-inflammatory molecules, a significant decrease in the anti-inflammatory marker IL-10 was noticed in the dysbiosis group as compared to eubiosis patients (*p* < 0.0001, Figure 4c).

Finally, the release of IGF-1, which has been related to eubiosis and endometrial proliferation [21], was analyzed in the same cohort. Our data demonstrated a significantly higher IGF-1 concentration in the eubiosis group than in the dysbiosis group (*p* = 0.0098, Figure 4d). These results demonstrate that patients with unbalanced endometrial microbiota are associated with higher pro-inflammatory molecules and lower anti-inflammatory markers.

Moreover, regression analyses demonstrated a clear relationship between the levels of inflammatory and inflammatory molecules in endometrial biopsies (Table 5). In particular, IL-1β was positively correlated with IL-6 (*p* = 0.0175), COX2 (*p* = 0.0448) and IL-8 (*p* = 0.0224). In contrast, the concentration of the anti-inflammatory marker IL-10 was negatively related to IL-6 (*p* = 0.0031), IL-1β (*p* = 0.0046), HIF-1α (*p* = 0.0081) and COX-2 (*p* = 0.0031) and positively related to IGF-1 (*p* = 0.0445). Consistently, IGF-1 was negatively correlated with IL-6 (*p* = 0.0252) and HIF-1α (*p* = 0.0290).

### 3.4. Correlation between Microbiota Composition and Inflammatory Markers in Endometrial Biopsies

The percentage of the microbial species within endometrial biopsies was correlated to biochemical parameters by regression analyses (Table 5). Lactobacillus abundance was negatively related to IL-1β (*p* < 0.0001), IL-6 (*p* = 0.0235), HIF-1α (*p* = 0.0002) and COX-2 (*p* = 0.0132) concentrations. Consistently, the percentage of Lactobacillus was positively related to the levels of IL-10 (*p* < 0.0001) and IGF-1 (*p* = 0.0310).

Concerning pathogen species, Streptococcus abundance was found to be directly related to IL-1b concentration (*p* = 0.0155). Similarly, Gardnerella percentage positively correlated with IL-6 concentration (*p* = 0.0006).

### 3.5. Correlation between Microbiota Composition, Biochemical Markers and Clinical Parameters Related to IVF Outcome

When the total population of enrolled patients was considered, no significant correlations were found between Lactobacillus and the number of IVF attempts (*p* = 0.3554). Similar results were obtained by selecting patients with dysbiosis (*p* = 0.8350). Interestingly, when the eubiosis group was selected, the Lactobacillus abundance was inversely related to the number of IVF attempts (*p* = 0.0394).

No significant correlation was evidenced between IVF attempts and the concentration of inflammatory factors, either in the total population and in the dysbiosis group (Table 5). Of note, by selecting the eubiosis group (see Table 5), the number of IVF attempts was found to be strictly related to the levels of the inflammatory factors COX-2 (*p* < 0.0001), IL-1β (*p* = 0.0004) and HIF-1α (*p* = 0.0371).

## 4. Discussion

Herein, the uterine microbiota composition and selected components of the cytokine network were analyzed in women presenting repeated implantation failure (RIF). The aim of the present study was to outline the relationship between uterine microbiota, inflammation and IVF outcomes. The enrolled women, presenting RIF, were divided into *Eubiosis* and *Dysbiosis* groups, according to the percentage of *Lactobacilli* present in the endometrial biopsy (≥90% and <90%, respectively). The main findings were as follows: (i) the *Dysbiosis* group presented significantly higher concentrations of the inflammatory markers IL-6, IL-1β, HIF-1α and COX-2 with respect to women with *Eubiosis*; (ii) the levels of the anti-inflammatory/well-being factors, IL-10 and IGF-1, were lower in the *Dysbiosis* group as compared to *Eubiosis* patients; (iii) *Lactobacillus* abundance was negatively related to the concentrations of the inflammatory molecules (IL-1β, IL-6, HIF-1α and COX-2) and positively related to IL-10 and IGF-1; (iv) the presence of microbial pathogens (Streptococcus and Gardnerella) positively correlated with IL-1 and IL-6 concentrations. When the IVF outcomes were considered, we found that: (i) the number of good quality embryos was inversely related to the IL-6 concentration in women with endometrial eubiosis; (ii) the number of ET was found to be strictly related to the levels of the inflammatory factors COX-2, IL-1β and HIF-1α, and inversely correlated to IGF-1 concentration in the *Eubiosis* group. Globally, our findings evidenced a strict link between endometrial dysbiosis and an alteration of the cytokine network, which influences IVF outcomes.

Microbial homeostasis in the female genital tract is crucial to maintain a healthy environment: lactobacillus abundance seems to be prevalent both in the superior and inferior reproductive system and fundamental in counteracting the overgrowth of other microorganisms [22]. A reduced relative abundance of Lactobacillus is associated to the development of several pathologic conditions including cervical cancer [23], HIV acquisition [24], endometriosis [25], and clinical and subclinical endometritis [26] with negative consequences on embryo implantation during IVF procedures [27].

Herein, we enrolled 26 women with RIF and uncertain or unexplained infertility diagnosis who underwent endometrial biopsy. The presence of dysbiosis was ascertained by 16 S RNA- RT PCR [19], allowing us to characterize the endometrial microbiota and to obtain detailed information on the quantity and type of pathogens eventually causing endometritis.

Of note, the identification of plasma cells by histological biopsies was not used in parallel. Moreover, the existence of plasma cells is a non-specific marker of inflammation and its presence in the biopsies can be obscured by a mononuclear cell infiltrate, plasmacytoid stromal cells, abundant stromal mitoses, a pronounced pre-decidual reaction in the late secretory endometrium, menstrual features, or secondary changes because of exogenous progesterone treatment before the biopsy [28,29,30].

The *Dysbiosis* group, characterized by a non-lactobacillus dominant microbiota, includes three women with a complete absence of lactobacilli species. Moreover, the most abundant pathogen infections were ascribed to *Streptococcus* and or *Gardnerella* species, which have been widely associated with endometritis, even in subclinical forms [31].

Literature data suggest that a preserved bacterial balance can preserve a corrected cytokine network [15], in turn increasing the chances of embryo implantation and promoting reproductive success [32]. In our study, a panel of inflammatory markers were quantified in the same endometrial biopsies. Specifically, pro-inflammatory interleukins (IL-1β, IL-6 and IL-8) and factors (i.e., HIF-1α and COX-2) were quantified in biopsies of the two study groups. Our findings evidenced a marked increase in the levels of inflammatory markers (IL-1β, IL-6, HIF-1α and COX-2) when the microbiota homeostasis was altered. IL-1β is also responsible for augmented levels of COX-2, which is an enzyme associated with inflammation and tumorigenesis. It has been reported that endometrial stem cells are hypersensitive to the stimulating effect of cytokines, such as interleukin-1β (IL-1β), in terms of the overexpression of COX-2 [33]. In this regard, it had been demonstrated that a low-dose-aspirin administration during IVF procedure improved oocytes’ and embryos’ quality through the COX 1-2 activity inhibition [34]. In *Dysbiosis* patients, higher levels of HIF-1α were found, as well. In this sense, a harmful endometrial microbiota could lead to the production of toxins which, acting through the TLR4 receptor, induce an increase in HIF-1α [20]. HIF-1α, in turn, may be involved in a molecular mechanism of endometrial dysfunction, negatively affecting uterine receptivity and successful embryo implantation [35].

In contrast, IL-10 and IGF-1 levels were higher in the *Eubiosis* group compared to the *Dysbiosis* one. IL-10 has important anti-inflammatory properties: its downregulation is associated to an adverse influence on the endometrial perfusion and receptivity to the extent that it could be considered predictive of a positive IVF outcome [36]. IGF 1, instead, has proliferative, differentiative and metabolic effects and it has been associated with endometrial differentiation, as well. Estrogens stimulate IGF-I gene expression in the endometrium, and IGF-I is assumed to mediate an estrogenic action [37]. Moreover, IGF-I seems to play a crucial role in the follicular and embryonic development, and data suggest that it could be considered a marker of embryo quality and implantation [38]. At the same time, a non-inhibition of the IGF-1 pathway could lead to an uncontrolled endometrial proliferation with harmful consequences for health [37]. As the intestinal microbiota has been demonstrated to play an essential role in the synthesis of IGF-1 [39], it is possible that a dysbiosis condition can cause an uncontrolled production of the growth factor leading to fertility complications and chronic endometrial inflammation.

The transition into a receptive uterus that is needed to acquire receptivity and overcome implantation failure is based on the modulation of different cytokines, growth factors, transcription factors, and prostaglandins [40]. A huge amount of data support the hypothesis that the process of embryo implantation actually requires the generation of an inflammatory response and the recruitment of immune cells that are required for the maternal immune tolerance [40]. Consistently, IVF patients with RIF subjected to endometrial biopsy present a higher expression of pro-inflammatory cytokine/chemokine expression and immune cells, with a final improvement in embryo implantation [40]. In discussing and interpreting our data, it has to be underlined that an endometrial dysbiosis leads to the instauration of a persistent inflamed microenvironment, similarly to those evidenced in endometriosis, which in turn decrease the chances of embryo implantation [41]. Thus, it is reasonable to hypothesize that the pro-inflammatory cytokine milieu, pivotal for implantation [42], should occur specifically within the embryo implantation window.

In our hand, the concentration of pro-inflammatory factors, i.e., IL-1β, IL-6, HIF-1α and COX-2, were inversely related to *Lactobacillus* abundance, and consistently, the latter positively correlated with the levels of IL-10 and IGF-1. Concerning pathogen species, Streptococcus and Gardnerella abundance were significantly IL-1β and IL-6 concentrations, respectively. Globally, our data clearly evidenced that a *Lactobacillus*-dominant endometrial microbiota is pivotal to maintain a physiologically non-inflamed microenvironment. Consistent with our data, uterine and vaginal infections have been established as a triggering factor for a persistent inflammation and immune activation in the endometrium, finally impairing embryo implantation [7,43,44]. Overall, our data evidenced that an imbalance of microbiota composition in the uterine tract is linked to inflammatory processes, as reported in different gynecological conditions, including cancer and endometriosis [6,45,46].

Having established the link between uterine dysbiosis and inflammation, we then moved to analyze the relationship on IVF outcomes. The simple comparison between the number of IVF attempts did not differ between the two enrolled groups. Indeed, the objective of the study was to recruit women with RIF, which, for definition, include women who filed to conceive following two or three embryo transfer cycles, in which at least one good-quality embryo was transferred in each cycle [47,48]. Actually, patients with RIF comprise a heterogeneous group that can present with different clinical problems [48]: among these, we focused on the putative presence of endometrial dysbiosis and pathogens as actual causes of the RIF.

When the total population of enrolled patients was considered, no significant correlations were found between Lactobacillus and the number of good quality embryos or IVF attempts. Probably, the very low abundance of Lactobacillus in some of the enrolled patients does not allow the detection of the correlations evidenced in the *Eubiosis* subgroup. Indeed, when the *Eubiosis* group was selected, the *Lactobacillus* abundance positively correlated with the number of good quality embryos and was inversely related to the number of IVF attempts.

Consistent with our data, a dysbiotic endometrial microbiota profile has been recently confirmed to be associated with unsuccessful outcomes, whereas *Lactobacillus* is enriched in patients with live birth outcomes and higher pregnancy rates [49].

Moreover, by selecting the *Eubiosis* group, the number of IVF attempts was found to be strictly related to the levels of the inflammatory factors COX-2, IL-1β and HIF-1α. Accordingly, the number of ET was inversely correlated to the IGF-1 concentration. These data suggest that an endometrial microbiota disorder causes environment changes involving inflammation, which impair IVF outcomes, which are feeding episodes of RIF [50].

Of note, following the diagnosis of pathogens-caused dysbiosis, patients underwent antibiotic treatment and probiotic supplementation [51], according to their individual microbial conditions, in order to modify the altered uterine composition [51]. Five of these patients achieved a clinical pregnancy in the IVF attempt successive to dysbiosis treatment, therefore identifying dysbiosis as the cause of the RIF. The endometrial biopsies following antibiotic/probiotic supplementation showed a reestablishment of endometrial microbiota. However, at the moment, the low number of cases in the patients’ follow-up did not allow us comparing inflammatory molecules. Consistent with our data, when antibiotics and prebiotics/probiotics have been administrated in conditions of non-*Lactobacillus*-dominated microbiota, a significant improvement of the embryo implantation rate was achieved in a *Lactobacillus*-dominated microbiota [1,10].

In analyzing our results, the study limitations should be underlined. Among these, first of all, the low number of patients, due to the particularly of the enrolled patients: women with RIF, with uncertain infertility and no apparent sign of endometritis. Certainly, the investigation of additional parameters related to inflammation and immune system activation (such as macrophages recruitment, [50]) can allow establishing how pathogens-induced dysbiosis is able to modify the local endometrial microenvironment. Moreover, new knowledge can surely come by comparing our data with the endometrial microbiota of females who do not suffer from infertility, even if this protocol would be of difficult application, because these women have no indication for a endometrial biopsy. Nevertheless, our study is ongoing to follow-up with the patients with endometrial pathogens/dysbiosis who are following an antibiotic/probiotic regimen. These data will allow the unveiling of the impact of antibiotic/probiotic supplementation on the final outcome of IVF techniques in patients presenting RIF.

## 5. Conclusions

Our data showed that women with RIF presenting a non-*Lactobacillus* dominant microbiota exhibited higher concentrations of the inflammatory markers and lower levels of the anti-inflammatory/well-being factors with respect to women with *Eubiosis*, particularly regarding their *Lactobacillus* abundance. The degree of inflammation (i.e., the levels of the inflammatory factors) was inversely related to *Lactobacillus* abundance and in turn increased in the presence of endometrial pathogens. The concentrations of analyzed cytokines were linked to the process of embryo implantation, influencing the number of ET in patients with RIF. Overall, our data contribute to the identification of endometrial dysbiosis as a cause of inflammation-related endometrial changes affecting the process of embryo implantation. Moreover, this study underline the importance of assessing the uterine microbiota in infertile patients [43], even not presenting a symptomatic/clinic endometritis, and suggest the use of probiotic supplements to re-establish endometrial eubiosis. Indeed, while there is abundant evidence concerning an altered genital microbiota to elevated inflammation, understanding the risk factors and mechanisms through which it affects genital health, and in particular fertility, is essential in the management of IVF.

## Figures and Tables

**Table 1:** Nucleotide sequences and annealing temperature of the primers utilized in PCR experiments.

	Reverse	Forward	T Annealing
*16S*	AGGGTTGCGCTCGTTG	GTGCCAGCAGCCGCGGTAA	64 °C
*Lactobacillus*	CCACCTTCCTCCGGTTTGTCA	AGGGTGAAGTCGTAACAAGTAGCC	60 °C
*Enterobacteriaceae species*	CAGGTCGTCACGGTAACAAG	GTGGTTCAGTTTCAGCATGTAC	65 °C
*Enterococcus species*	AGAGAGTAAGGTCCGATTGAAC	GGTTGTTTCCCGTATTATGC	55 °C
*Escherichia coli*	AGAAGCTTGCTCTTTGCTGA	CTTTGGTCTTGCGACGTTAT	59.3 °C
*Gardnerella vaginalis*	TTACTGGTGTATCACTGTAAGG	CCGTCACAGGCTGAACAGT	57 °C
*Klebsiella pneumoniae*	ACGGCCGAATATGACGAATTC	AGAGTGATCTGCTCATGAA	56 °C
*Staphylococcus species*	CAGGAGAAGTTAAAGAACAAGAAG	GTGAACGAACTAATTGAGATACG	58.4 °C
*Streptococcus species*	GTACAGTTGCTTCAGGACGTATC	ACGTTCGATTTCATCACGTTG	55 °C
*Pseudopropionibacterium*	TGCTTTCGATACGGGTTGAC	AGGAGGTGATCCAACCGCA	60 °C
*Prevotella*	GTGGCGCGTATTTTATGTATGTG	ATCCGCCATACGCCCTTAG	59 °C
*Neisseria Subflava*	CCAACGATGTTCGCGAATTG	TGGAAGACGGATTTGGTGTAAT	58 °C
*Mycoplasma hominis*	CATGCATGTCGAGCGAGGTT	CCATGCGGTTCCATGCGT	57 °C
*Bifidobacterium*	ATCGCAGTCTGCAACTCGA	ATCCGAACTGAGACCGGTT	59 °C
*Neisseria gonorrhoeae*	GTTTCAGCGGCAGCATTCA	CCGGAACTGGTTTCATCTGATT	57 °C
*Chlamydia trachomatis*	GGATCCGTAAGTTAGACGAAATTTTG	TTTAATGCGAAAGGAAATCTGATTG	58.1 °C

**Table 2:** Descriptive statistics of clinical parameters for the two study groups. The data are expressed as mean ± SD. Good quality embryos, number of IVF cycles, number of embryo-transfer (ET), good quality embryos/number of trials are expressed as median values. AMH: anti-Mullerian hormone; ET: embryo-transfer; IVF: in vitro fertilization; BMI, body mass index.

Parameter	*Eubiosis* Patients	*Dysbiosis* Patients	*p*-Value
Age (years)	38.5 ± 4.4	39.1 ± 2.7	0.7511
Occupation (N)	11	10	
BMI	21 ± 2.4	22.0 ± 1.7	0.4112
Years of infertility	4.2 ± 1.4	3.9 ± 1.1	0.5492
AMH (ng/mL)	2.1 ± 1.1	1.9 ± 0.9	0.6471
IVF cycles (N)	2	2	>0.9999
Good quality embryos (N)	5.00	4.5	0.8524
Good quality embryos/number of trials	2	2.667	0.4951
Number of ET	4	4	>0.9999

**Table 3:** Descriptive statistics of microbial parameters for the two study groups. The data are expressed as mean ± SD. Statistical analysis was performed by unpaired *t*-test. **** *p* < 0.0001 vs. eubiosis patients.

Endometrial Species	*Eubiosis* Patients (Composition of Microbial Species % versus Total)	*Dysbiosis* Patients (Composition of Microbial Species % versus Total)
Lactobacillus	97.9 ± 3.7	27.3 ± 21.8 ****
Streptococcus	8.00 ± 0.50	49.78 ± 39.37
Staphylococcus	7.78 ± 1.20	7.78 ± 0.90
Bifidobacterium	0	39.7 ± 37.5
Gardnerella	0	50.6 ± 14.0
Propionibacterium	6.4 ± 1.2	6.4 ± 1.2

**Table 4:** Biochemical parameters measured in the two study groups. The data are expressed as mean ± SD. Statistical analysis was performed by unpaired *t*-test. * *p* < 0.05, ** *p* < 0.01, *** *p* < 0.001 **** *p* < 0.0001 vs. eubiosis patients.

Biochemical Parameters	*Eubiosis* Patients (MEAN ± SD)	*Dysbiosis* Patients (MEAN ± SD)
IL-1β pg/µg	28.5 ± 9.6	73.0 ± 28.8 ****
IL-6 pg/µg	6.43 ± 7.85	12.7 ± 6.3 *
IL-8 pg/µg	2203 ± 843	2321 ± 659
IL-10 pg/µg	58.0 ± 8.8	30.4 ± 10.2 ****
HIF-1α ng/µg	7.58 ± 4.27	16.9 ± 5.9 ***
IGF-1 ng/µg	39.9 ± 25.2	14.5 ± 11.8 **
COX-2 ng/µg	24.9 ± 12.2	43.2 ± 21.0 *

**Table 5:** Correlation between microbiota composition, biochemical markers and clinical parameters related to Total population, in eubiosis and dysbiosis subjects; Z and *p* values obtained for each correlation are reported in the respective column. ET, embryo-transfer.

	Total Population			*Eubiosis* Patients			*Dysbiosis* Patients		
	Correlation	Z-Value	*p*-Value	Correlation	Z-Value	*p*-Value	Correlation	Z-Value	*p*-Value
Lactobacillus, IL-1β	−0.776	−4.784	<0.0001	−0.514	−1.706	0.0880	−0.455	−1.534	0.1251
Lactobacillus, IL-10	0.767	4.641	<0.0001	−0.033	−0.098	0.9221	0.086	0.273	0.7851
Lactobacillus, HIF-1α	−0.705	−3.824	0.0001	0.377	1.189	0.2346	−0.306	−0.893	0.3716
Lactobacillus, IGF-1	0.457	2.210	0.0271	0.108	0.327	0.7439	−0.439	−1.413	−0.809
Lactobacillus, COX-2	−4.494	−2.480	0.0132	0.562	1.908	0.565	−0.239	−0.760	0.4473
Lactobacillus, Good quality embryos	0.041	0.174	0.8618	−0.642	−2.285	0.0223	−0,25	−0.066	0.9476
Lactobacillus, Number of trials	0.214	0.924	0.3554	−0.630	−2.223	0.0262	−0.079	−0.208	0.8350
IL-1β, IL-6	0.476	2.376	0.0175	−0.602	−2.087	0.0369	0.751	3.088	0.0020
IL-1β, IL-10	−0.540	−2.833	0.0046	0.594	2.051	0.0403	0.058	0.182	0.8553
IL-1β, HIF-1α	0.551	2.073	0.0069	−0.310	−0.963	0.3354	0.117	0.331	0.7405
IL-1β, COX-2	0.404	2.007	0.0448	−0.646	−2.307	0.0210	0.197	0.630	0.5286
IL-1β, Good quality embryos	−0.151	−0.661	0.5085	0.701	2.609	0.0091	−0.560	−1.673	0.0944
IL-1β, Number of trials	−0.090	−0.394	0.6934	0.845	3.719	0.0002	−0.007	−0.019	0.9849
IL-6, IL-8	0.481	2.283	0.0224	0.560	1.900	0.0574	0.292	0.851	0.3949
IL-6, IL-10	−0.568	−2.955	0.0031	−0.838	−3.640	0.0003	−0.123	−0.390	0.6965
IL-6, IGF-1	−0.462	−2.238	0.0252	-0.360	−0.949	0.3426	−0.198	−0.602	0.693
IL-8, HIF-1α	0.235	0.987	0.3237	0.658	2.366	0.0180	0.322	0.817	0.4141
IL-8, COX-2	0.136	0.596	0.5515	0.599	2.077	0.0378	0.245	0.707	0.4794
IL-10, HIF-1α	−0.542	−2.647	0.0081	0.099	0.298	0.7653	0.078	0.221	0.8253
IL-10, IGF-1	0.412	2.010	0.0445	0.247	0.756	0.4494	−0.289	−0.894	0.3715
IL-10, COX-2	−0.558	−2.956	0.0031	0.096	0.290	0.7721	−0.465	−1.593	−0.809
HIF-1α, IGF−1	−0.474	−2.183	0.0290	−0.566	−1.926	0.0542	0.530	1.561	0.1184
HIF-1α, COX-2	0.430	2.005	0.0449	0.552	1.864	0.0623	−0.172	−0.492	0.6228
HIF-1α, Good quality embryos	0.166	0.671	0.5023	−0.605	−2.103	0.0355	0.697	1.926	0.0541
HIF-1α, Number of ET	−0.393	−1.662	0.0965	0.848	3.750	0.0002	−0.408	−1.224	0.2208
IGF-1, Good quality embryos	−0.240	−1.066	0.2862	−0.597	−2.067	0.0388	0.061	0.163	0.8706
Cox-2, Number of ET	0.022	0.097	0.9230	−0.924	−4.839	<0.0001	−0.430	−1.453	0.1462

## Data Availability

Data are contained within the article.
